# Craving Behavior Intervention in Ameliorating College Students' Internet Game Disorder: A Longitudinal Study

**DOI:** 10.3389/fpsyg.2017.00526

**Published:** 2017-04-10

**Authors:** Lin-Yuan Deng, Lu Liu, Cui-Cui Xia, Jing Lan, Jin-Tao Zhang, Xiao-Yi Fang

**Affiliations:** ^1^Faculty of Education, Beijing Normal UniversityBeijing, China; ^2^Faculty of Psychology, Institute of Developmental Psychology, Beijing Normal UniversityBeijing, China; ^3^Students Counseling Center, Beijing Normal UniversityBeijing, China; ^4^State Key Laboratory of Cognitive Neuroscience and Learning and IDG/McGovern Institute for Brain Research, Beijing Normal UniversityBeijing, China; ^5^Center for Collaboration and Innovation in Brain and Learning Sciences, Beijing Normal UniversityBeijing, China

**Keywords:** craving behavior intervention, internet gaming disorder, college students, active ingredients, depression, psychological needs

## Abstract

Craving, as a central feature of addiction and a precursor of relapse, is targeted recently in addiction intervention. While Internet gaming disorder (IGD), conceptualized as a behavioral addiction, is lack of effective treatment practice and exploration of its mechanism. This research aims to test the effectiveness and detect the active ingredients of craving behavior intervention (CBI) in mitigation of IGD among young adults. A total of 63 male college students with IGD were assigned into the intervention group (six-session CBI intervention) or the waiting-list control group. Structured questionnaires were administered at pre-intervention (T1), post-intervention (T2), 3-month follow-up (T3), and 6-month follow-up (T4). Compared to the control group, a significant decrease in the severity of IGD in intervention group was found at post-intervention and lasting to 6 months after intervention. The value changes of craving could partially mediate the relationship between intervention and changes of IGD among all effects tests (immediate, T2-T1; short-term, T3-T1; and long-term effects, T4-T1). Further, explorations of the active ingredients of intervention found depression relief and shift of psychological needs from Internet to real life significantly predict craving amelioration at both post-intervention and 6-month follow-up. Although preliminary, the current study provides evidence for the value of craving-aimed intervention practice in IGD treatment and identifies two potential active ingredients for mitigation of craving, and the long-term therapeutic benefits are further conferred.

Registry name: The behavioral and brain mechanism of IGD;

URL: https://www.clinicaltrials.gov/ct2/show/NCT02550405;

Registration number: NCT02550405.

## Introduction

Internet gaming has become an indispensable spare-time activity for adolescents as well as adults (PC Gaming Alliance, 2013; China Internet Network Information Center, 2016). Specifically, in 2012, more than one billion individuals played games around world (PC Gaming Alliance, 2013). By 2015, there were 0.38 billion Internet game users in China, among which mainly adolescents and young adults with age from 10 to 29 (China Internet Network Information Center, 2016). Internet gaming disorder (IGD), with the morbidity of IGD about 8–13.7% in mainland (Block, 2008; Cao et al., 2011), and 46% in Taiwan area (Wan and Chiou, 2006), as the most prevalent subtype (57.5%) of Internet addiction disorder (IAD; Chen et al., 2014), is defined as persistent and recurrent use of the Internet to engage in games (American Psychiatric Association, 2013). Given the growing prevalence and negative consequences (e.g., poor academic performance, impaired social interaction, dysfunctional cognitive functioning, low well-being and high loneliness; psychosomatic disorder; Kuss, 2013), IGD recently has been included in the fifth edition of the Diagnostic and Statistical Manual of Mental Disorders (DSM-5) as a disorder warranting further study (American Psychiatric Association, 2013). Thus, further research is needed in the development of more effective interventions.

Evidence from substance use disorder (SUD) revealed that, as a motivational state associated with a strong desire to drug taking, craving plays an important role in directly targeting compulsive drug use and relapse (Tiffany and Wray, 2012), which also has been supported by recent data demonstrating the mediate effects of craving on treatments and outcomes (Ferguson and Shiffman, 2009; Witkiewitz et al., 2011) and its neural underpinnings (Kober et al., 2010; Sinha, 2013; Westbrook et al., 2013).

For behavior addiction, evidence from gambling disorder (GD) implicated craving a core component (Potenza, 2008), its role played in assessing treatment outcomes (Grant et al., 2003) as well as its role as a treatment target (Kim and Grant, 2001). Treatment research in GD refers to both psychotherapeutic and pharmacological approaches with the later predominant (Brewer et al., 2008; Leeman and Potenza, 2012; Yip and Potenza, 2014). Among pharmacotherapies, preliminary studies have been performed into relationships among craving/impulsivity, and treatment outcome to test the efficacy of opioid antagonists/serotonin reuptake inhibitors, with the opioid antagonists having more consistent efficacy in reduction of craving and addiction severity (Brewer et al., 2008; Leeman and Potenza, 2012). Additionally, multiple behavioral therapies have demonstrated the effects in treating GD symptoms (e.g., gambling urges) and gambling behaviors (Brewer et al., 2008; Yip and Potenza, 2014).

As for IGD, gaming craving has been enhanced and demonstrated a central critics of IGD diagnosis (Ko et al., 2014), a component of addictive behaviors (Kuss and Griffiths, 2012), while also its neural substrates underlying (Ko et al., 2009a, 2013; Liu et al., 2016) and index of treatment outcomes (Han et al., 2010, 2012). However, whether craving might play its role as clinical target for treatment in IGD remain to be investigated.

According to existed interventional researches in IGD, approaches mainly focus on psychotherapeutic and pharmacological interventions with the former predominant (Winkler et al., 2013; King and Delfabbro, 2014). Psychotherapeutic intervention practices existed mainly focus directly on symptoms, gaming behaviors and cognitions related to Internet gaming (Winkler et al., 2013; King and Delfabbro, 2014), with no reference to factors related to addiction and relapse such as craving. Although some previous pharmacological intervention practices have demonstrated effects of medications on alleviating online gaming cravings (Han et al., 2010; Kim et al., 2012), the underlying mechanisms haven't been revealed up to date. Next, as a common limitation of the past interventional researches, only three of the whole eleven studies (Du et al., 2010; Su et al., 2011; Kim et al., 2012) conducted a follow-up assessment, with the follow-up periods ranging from 1 month (Su et al., 2011) to 6 months (Du et al., 2010; Kim et al., 2012). Since relapse is a crucial indicator of intervention outcome (King and Delfabbro, 2014; Sayette, 2016), long-term follow-up tracking is of great importance. Furthermore, since the focus of interventional research is increasingly on the mechanisms of behavioral changes (Longabaugh and Magill, 2011), the investigation of active treatment ingredients, which can lead to increased understanding of the condition, is of importance in further demonstrating mechanisms of specific treatment interventions. Thus, active ingredients and operative change mechanisms of the craving behavior intervention (CBI) will be explored.

### About craving

Craving, defined as a drug acquisitive state motivating drug use, has long been a focus of study as its central feature in the field of addiction (Sayette, 2016). Since the twenty-first century, thousands of researches have been published on craving (Tiffany and Wray, 2012), which, to some extent, may lead to the recent inclusion as diagnostic criterion for substance use disorders in the fifth edition of the Diagnostic and Statistical Manual Disorders (DSM-5; American Psychiatric Association, 2013).

Craving plays a key role in predicting and driving addictive behavior (Serre et al., 2015) and relapse (Baker et al., 2006), which has been demonstrated in a large number of studies including intervention practices (Sayette and Tiffany, 2013). Similarly, in the field of IGD, studies have also confirmed that the criterion of craving had diagnostic accuracy of 88% to differentiate university students with IGD from remitted students (Ko et al., 2014), and that bupropion sustained release treatment, which has been found to alleviate drug craving, significantly decreased total game play time and activities of cue-induced- related brain regions (Han et al., 2010).

For the mechanism of craving-aimed intervention, there exist two causal pathways: craving reactivity-reduction in a bottom-up manner and craving regulation in a top-down manner (Sinha, 2013; Westbrook et al., 2013). The former refers to directly decrease of craving reactivity and neural circles underpinning, by attenuating the negative affective states, compulsive “wanting” states, or weakening the association between addiction-related cue-exposure and craving or drug-seeking. As for the top-down pathway, a large body of work has identified the disruption of inhibitory control over behavioral approach (Sayette and Creswell, 2016), which has been demonstrated by neural evidence from craving regulation intervention (Kober et al., 2010). To improve self-control and regulation of craving may in the long run decrease craving circuits' reactivation that trigger approaching behaviors (Sinha, 2013). Since no previous researches, to our knowledge, have involved both components of craving- attenuating and inhibitory control strategies within an intervention practice, the potential effects of the two pathways on craving reduction is not clear.

### The bottom-up pathway

#### Craving and emotion

According to models outlined previously (Baker et al., 2004), addiction is developed by associative learning mechanisms and perpetuated through negative reinforcement. The well-established associative memories between negative (e.g., when “stressed”) affective states and substance use among individuals with SUD can trigger craving to substance (Westbrook et al., 2013), and relapse (Skinner and Aubin, 2010). The correlation between negative affect and gambling craving has also been revealed in GD (de Castro et al., 2007), and the negative affect along with craving as treatment outcome have been demonstrated the sensitivity to pharmacological treatment (Fong et al., 2008). Accumulating evidence suggests co-occurrence of IGD and negative affective experience (e.g., depression; Meng et al., 2014; Zhang et al., 2015; Yao et al., 2017), with a preliminary intervention using mindfulness meditation found its effect in reduction of negative affect (Yao et al., 2017). Further, evidence from intervention indicates that mindfulness-based interventions have been demonstrated efficacy for craving reductions mainly through alleviating negative emotional states (e.g., depression and anxiety; **?**Westbrook et al., 2013), and through weakening the relationship between negative emotional states, craving and addictive behaviors (Witkiewitz and Bowen, 2010; Witkiewitz et al., 2011).

#### Craving and psychological needs

Psychological need is considered one of the most crucial driving forces that promote behavioral change (Liu et al., 2015). Considering the overwhelming internal motive of adolescents with Internet addiction, fulfillment of psychological needs through Internet use has been proposed (Suler, 1999; Merrill and Christine, 2006), leading to the dominant routine desire among IAD subjects at the expense of normal psychological needs. An intervention aiming at craving reduction through enhancing family cohesion and affection (adolescents' psychological needs for relatedness) has observed both decrease in Young Internet Addiction Scale (YIAS) scores and improvement in family cohesion (Han et al., 2012). Another study with multi-family group therapy (MFGT) to reduce Internet addiction among adolescents has reported that the decrease in adolescent Internet use was partially explained by the improved parent–adolescent communication and closeness (Liu et al., 2015).

### The top-down pathway

#### Craving and time-management

Failure in self-regulation appears to extend the subjective experience of time which also appears to pass more slowly in individuals with craving (Vohs and Schmeichel, 2003; Sayette et al., 2005). For instance, Internet and Facebook related stimuli could distort time perception due to attention and arousal related mechanisms (Gonidis and Sharma, 2017). For gaming disorders, excessive time spending on Internet gaming as a particular feature has been referred in five of the nine criteria for IGD in the DSM-5 (American Psychiatric Association, 2013), and time-management has been implicated as one of the main factors underlying negative consequences of IGD (Chen et al., 2003). Besides, according to the theory of planned behavior (Ajzen, 1991), a model of attitude-behavior relationships, individual's intention is strongly related to a given behavior. Under volitional control, motivations combined with perceived behavioral control (the likelihood of behavioral achievement), as components of intention, would be expected to influence performance. Thus, through reassigning individual Internet using schedule and in comparison with actual Internet using time, a strategy might help regulating craving through awareness of attitude-behavior conflict, as well as enhancing motivation and self-efficacy for abstinence.

#### Craving and impulse control

Impulsivity, implicated in diagnostic criteria “loss of control” and “continued engagement despite negative consequences” for both SUD and GD (American Psychiatric Association, 2013), is a core component of addiction (Potenza, 2008), and there is no exception for IGD (Petry et al., 2014). Studies among IGD participants have implicated the relationship between dysfunction of inhibition control and addictive behaviors (Meng et al., 2014; Yao et al., 2015), and preliminarily demonstrated the effects of combined reality therapy and mindfulness meditation in reduction of decisional impulsivity (Yao et al., 2017). Further, evidence from neural studies highlighted the imbalance between the brain cognitive system and reward system in addiction (Volkow and Baler, 2014), which lead to inability of the individual to suppress aggressive thoughts and impulsive aggressive behavior (George and Koob, 2010). Coping strategies training associated with addiction might prompt individuals' impulse control by altering their focus to future-oriented goals (Potenza et al., 2011), and have been shown to reduce craving as well as relapse, and related brain activations (Kober et al., 2010). Thus, impulse control is hypothesized another modulator for both regulation of craving and addiction behaviors.

In the current study, a CBI was developed, which was tailored to releasing depression experience, shifting young adults' fulfillment of psychological needs from the Internet to real life, coping skills learning of gaming-time management and impulse control, aiming at systematically investigating the effects of this behavioral intervention practice on IGD, tracking long-term follow-up of the effects, as well as detecting active ingredients of intervention. Based on previous findings mentioned, we hypothesized that: (1) the intervention group shows a reduction in self-reported craving and severity of IGD at the end of the intervention and at 3- and 6-month follow-up compared with the control group; and the effectiveness of the intervention can be explained by the change of craving; (2) participants in the intervention group show improved depression release, psychological need satisfaction in real life, gaming-time management and impulse control; (3) the improved depression release, psychological need satisfaction in real life, gaming-time management and impulse control could largely explain the change of craving, and also directly explain the change of IGD.

## Materials and methods

### Participants

Sixty-three individuals with IGD were recruited via the Internet and advertisements posted at local universities and selected through an online questionnaire and telephone screening, and included in the intervention practice. Given the extremely higher prevalence of IGD in men vs. women (Ko et al., 2009b), only male participants were included.

**Figure 1:**
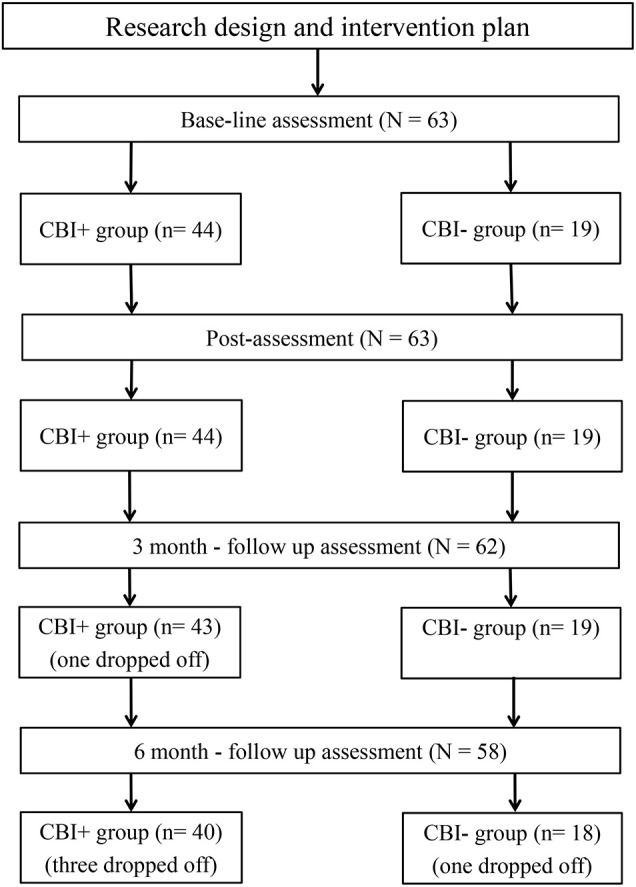
**The intervention process and participants flow diagram**.

Participant assignment was according to their willingness and curriculum arrangement. Forty-four of the 63 IGDs were assigned in the CBI intervention (CBI+ group, into one of 5 therapy groups), the other 19 IGDs were included in the control group (CBI− group) with 11 of them had no relevant spare-time and the rest non-treatment seeking then. Individuals in the control group were added to the waiting-list for the intervention after the informed consent. (See Figure 1 for the intervention process and participants flow diagram).

Participants were recruited according to their weekly Internet gaming time and scores on the Chen Internet Addiction Scale (Chen et al., 2003). Inclusion criteria for IGDs were: (1) a score of 67 or higher on the CIAS (Ko et al., 2009b); (2) engagement in Internet gaming for over 20 h per week for a minimum of 1 year; and (3) reporting of Internet gaming as their primary online activity (Yao et al., 2014, 2015). Exclusion criteria were assessed through a semi-structured personal interview to exclude the candidate fulfilling DSM-5 criteria for abuse or dependence of substances, including alcohol. Further, participants who reported current or history of use of illegal substances and any gambling experience (including online gambling) were excluded. Additionally, any self-reported history of any psychiatric or neurological illness, as well as current use of any psychotropic medication was excluded (Yao et al., 2015).

Tobacco-use characteristics were assessed using the Fagerstrom Test for Nicotine Dependence (FTND; Fagerstrum, 1978), and nicotine-dependent individuals were excluded (i.e., individuals with an FTND score ≥6; Fagerstrom et al., 1990). Alcohol consumption was assessed using the Alcohol Use Disorder Identification Test (AUDIT-C; Bush et al., 1998), and participants with AUDIT-C scores ≥5 (Dawson et al., 2005) were instructed to complete the Michigan Alcoholism Screening Test (MAST; Selzer, 1971) for further screening. Individuals with a score ≥6 on the MAST were excluded for alcohol dependence. Current depression and anxiety symptoms were assessed using the Beck Depression Inventory (BDI; Beck et al., 1961) and the Beck Anxiety Inventory (BAI; Beck et al., 1988), respectively.

This study was approved by the Institutional Review Board of the School of Psychology, Beijing Normal University. All participants provided written informed consent and were financially compensated for their time.

### Intervention

The CBI was a face-to-face group therapy program, given once a week for 6 weeks, conducted by four therapists with similar clinical background in behavioral therapy and group therapy. A pair of therapists was randomly assigned to a CBI+ group. The 44 IGD participants under intervention were divided into five groups with 8–10 persons each group. Among them, three groups were conducted by a couple of therapists, while the other two group were intervened by another couple of therapists (see Table S1). Each session included 5 parts in 2.5–3 h: a warming-up exercise, a discussion about the homework from the last session (except the first session), a main structured activity, a brief summary, and the homework assignment.

The topics for each session focused on: (1) session 1, understanding and perceiving subjective craving for Internet gaming, listing gaming-related scenes that might trigger craving; (2) session 2, recognizing and testing irrational beliefs on craving and exploring other possible inferences; (3) sessions 3, detecting emotions triggering craving and searching effective experience of regulation; (4) session 4, shifting participants' fulfillment of psychological needs from the Internet to reality and building adaptive relationships with peers; (5) session 5, time management and skills training for coping with craving; (6) session 6, maintaining the effectiveness of the intervention through reviewing and summarizing the CBI, and setting up adaptive and positive plans for daily life in the future. Each session has mindfulness training related to the focus of each session. In addition, mindfulness training was self-administered every time they experienced craving outside the intervention hours as a homework assignment.

### Measurement

#### Internet addiction disorder

The Chinese Internet Addiction Scale (CIAS), designed to assess the severity of Internet use and addiction, consists of 26 items related to tolerance, withdrawal, compulsive Internet use, time management, and interpersonal relationships problems and healthy problems (Chen et al., 2003). This study utilized CIAS mainly for participants screening. Each item uses a scale from 1 (Strongly disagree) to 4 (Strongly agree), and the CIAS total score is calculated by the sum of the items. The reliability and validity of the CIAS among college students has been demonstrated previously (Chen et al., 2003). The Cronbach's alpha in this study was 0.88.

#### Internet gaming disorder (IGD)

The problematic online game use scale (POGUS), a measure of people's excessive use of online games which results in negative outcomes (i.e., psychological, social, school, and work difficulties in a person's life; Min and Kim, 2010). The POGUS consists of 20 items with the rating for each item ranges from 1 (totally disagree) to 5 (totally agree), and the total score is calculated by the sum of the items. A Chinese adaption of the scale has been used in a previous study (Zhang et al., 2012), with the Cronbach's alpha for the scale of 0.92 and the retest reliability of 0.75. The Cronbach's alpha in this study was 0.89.

#### Craving for online gaming (VAS)

According to previous studies about subjective craving for substance use and online gaming, a single-item scale was conducted to participants about how much they craved for online game now (Tiffany et al., 2000; Ko et al., 2009a). The rating of the scale ranges from 1 (not at all) to 7 (extremely extensive craving), the higher the score the more craving subject feels then.

#### Beck's depression inventory

Depression was assessed using a Chinese version of the Beck's depression inventory - second edition (BDI-II; Beck et al., 1961; Wang et al., 2011), a measure consisting of 21 multiple-choice format items assessing specific symptoms of depression over the past 2 weeks, with each answer given a score between 1 and 4. The total BDI score is calculated by summing scores for items. The Cronbach's alpha was 0.88 in this study.

#### Online gratification of psychological need (Psy-needs)

A modified college students' psychological need and gratification questionnaire (CSPNIGQ, Wan et al., 2010) was conducted to assess participants' gratification of psychological needs from reality vs. from the Internet. Combining two of the CSPNIGQ's three subscales: reality gratification questionnaire and the Internet gratification questionnaire with the original structure, the modified scale includes 44 items that tap into eight kinds of need: need for power, identity, meeting challenge, social (interpersonal interaction), avoiding reality, autonomy, cognition and achievement. The rating for the items ranges from 1 (mainly satisfied from real life) to 5 (mainly satisfied from Internet). The questionnaire has good structure validity, consistency, split-half and retest reliability. The CFA shows that the model is best fit the data and has rational structure. The total score is calculated by the sum of the items, and the higher the participant's score the higher the Internet gratification of psychological needs. The Cronbach's alpha was 0.93 in this study.

#### The proportion of internet gaming play among internet use

Participants reported the hours they spent on Internet using (with Internet gaming hours included) and the hours they spent on Internet gaming per week. And the proportion they spent on gaming was calculated from dividing their gaming hours by the entire time they spent on the Internet.

#### Barratt impulsivity scale version 11 (BIS-11)

The Chinese version of the BIS-11 (Patton et al., 1995; Li et al., 2011) contains 30 self-administered items that designed to measure impulsiveness, including attention impulsiveness, motor impulsiveness, and non-planning impulsiveness. All items are answered on a 4-point scale (“1” refers to rarely/never, “2” refers to occasionally, “3” refers to often, and “4” refers to almost always/always) with 4 indicates the most impulsive response. The higher the summed score for all items, the higher the level of impulsiveness. The Cronbach's alpha was 0.54 in this study.

### Research procedure

The procedures of this study were as follows: (1) Craving Behavioral Intervention was developed based on the theoretic framework of behavioral therapy, group therapy, previous intervention practices, and empirical studies. A pilot study was conducted among 8 individuals with IGD before the intervention was launched, to assess the validity of the intervention and modify potential problems. (2) Recruited participants were divided into intervention (CBI+) & control (CBI−) groups according to their schedule and willingness, and provided informed consent for their participation. (3) The CBI+ group were asked to complete assessments before (T1) and after the intervention (T2), at a 3-month (T3) and 6-month (T4) follow-up as well, with the CBI- group to complete four sessions of assessments at the same time points. (4) All participants were paid ¥100 for their participation. The details of the procedures are presented in Figure 1.

## Results

### Effectiveness of the intervention

Demographic characteristics and Internet-related measures at baseline (T1) were compared between the CBI+ and the CBI− group with no significant group difference identified, indicating that the two groups were at the similar level in age, education and severity of Internet gaming disorder. According to AUDIT-C, 34 of the 44 CBI+ participants and 13 of the 19 CBI− participants were occasional alcohol drinkers (non-dependent drinkers). None of the participants met criteria for alcohol dependence, as defined by a score ≥5 on the AUDIT-C. Three CBI+ participants and 1 CBI− reported occasional cigarette smoking (see Table 1).

**Table 1:** **Demographic characteristics between CBI+ and CBI− group**.

**Variable**	**CBI+**	**CBI−**	***t/χ2*** **value**
	***M (SD)***	***M (SD)***	
Age	21.86 (1.90)	22.05 (1.81)	−0.37
Education level	15.8 (1.73)	15.21 (1.78)	1.21
Severity of IGD (POGUS)	77.68 (11.48)	73.68 (8.18)	1.37
BAI	8.52 (9.09)	8.17 (7.19)	0.58
BDI	33.23 (8.28)	30.83 (4.59)	1.41
Cigarette use (FTND)	3 (6.82%)	1 (5.26%)	0.55
Score of FTND	1.33 (1.15)^a^	0.00^b^	–
Alcohol use (AUDIT-C)	34 (77.27%)	13 (68.42%)	0.05
Score of AUDIT-C	1.79 (1.81)^c^	1.54 (1.13)^d^	0.48

*SD, standard deviation; CBI+, subjects with Internet gaming disorder who received craving behavioral intervention; CBI−, subjects with Internet gaming disorder who did not receive craving behavioral intervention; POGUS, the problematic online game use scale; BAI, Beck Anxiety Inventory; BDI, Beck Depression Inventory; FTND, Fagerstrom test for nicotine dependence; AUDIT-C, Alcohol use disorder identification test*.

a *n = 3*;

b *n = 1*;

c *n = 34*;

d *n = 13*.

Results from repeated measures ANOVA (see Table 2) showed a group (CBI+ & CBI−) by assessment (pre-post-intervention/assessment) interaction for the severity of IGD (score of POGUS) [*F*_(3, 54)_ = 9.08, *p* < 0.001], and the simple effect test showed significant difference across the four time-point measurements in the CBI+ group [F _(3, 53)_ = 64.76, *p* < 0.001], which indicates that the intervention effects maintained about 6 months after intervention. Furthermore, *post-hoc* test within the intervention group displayed significant decrease in the severity of IGD in Time 2 (T1–T2 = 21.11, *p* < 0.001), Time 3 (T1–T3 = 24.54, *p* < 0.001), Time 4 (T1–T4 = 24.42, *p* < 0.001) compared to the baseline-measure, and no significant differences were found between T2, T3, and T4. In the control group, simple effect tests showed significant difference across the four time-point measurements in severity of IGD [*F*_(3, 53)_ = 3.27, *p* < 0.05]. However, the score in control group is consistently higher than intervention group.

**Table 2:** **Comparisons of measured variables between the CBI+ and the CBI− group at T1, T2, T3, and T4**.

**Variable**	**Group**	**T1**	**T2**	**T3**	**T4**		***F*****^a^**	***F*****^b^**
		***M (SD)***	***M (SD)***	***M (SD)***	***M (SD)***			
IGD	IGD+	77.68 (11.48)	56.57 (8.53)	53.14 (11.91)	53.26 (12.03)		9.08^***^	64.76^***^
	IGD−	73.68 (8.18)	66.39 (11.23)	66.17 (16.57)	64.72 (13.02)			3.27^*^
	*t*	1.37	−3.80^***^	−3.39^***^^c^	−3.27^**^^d^			
VAS	IGD+	3.52 (1.23)	2.41 (1.06)	2.30 (1.01)	2.33 (0.94)		8.67^***^	10.84^***^
	IGD−	2.79 (1.55)	3.32 (1.38)	3.74 (1.69)	3.44 (1.54)			2.04
	*t*	2.00^*^	−2.84^**^	−4.14^***^	−3.40^***^^e^			

* *p < 0.05*;

** *p < 0.01*;

*** p < 0.001;

F^a^ *, the effects of group × time points interaction*;

F^b^ *, the simple effects of time points in each group*;

c *n = 43*;

d *n = 40*;

e *n = 18*.

Self-reported craving (score of VAS) also showed a group by assessment interaction [*F*_(3, 54)_ = 8.67, *p* < 0.001]. The simple effect test showed significant difference along the measurement-time in the CBI+ group [*F*_(3, 53)_ = 10.84, *p* < 0.001]. *Post-hoc* test within the CBI+ group displayed significant decrease in the severity of IGD in Time 2 (T1–T2 = 1.11, *p* < 0.001], Time 3 (T1–T3 = 1.22, *p* < 0.001), and Time 4 (T1–T4 = 1.19, *p* < 0.001) compared to the baseline-measure and no significant differences were found between T2, T3, and T4, which indicate that the intervention effects maintained after intervention. In control group, no significant difference was found in craving [*F*_(3, 53)_ = 2.04, *p* > 0.05; see Table 2].

### Mediation effects of craving regulation

To examine the immediate, short-term, and long-term effects of the craving-aimed intervention for the mitigation of IGD, three mediation models were conducted and tested. We set the group (intervention & control) as the independent variable, and the change values (immediate: ΔX = T1–T2; short-term: ΔX = T1–T3; long-term: ΔX = T1–T4) of craving and severity of IGD as mediator and dependent variable respectively.

**Figure 2:**
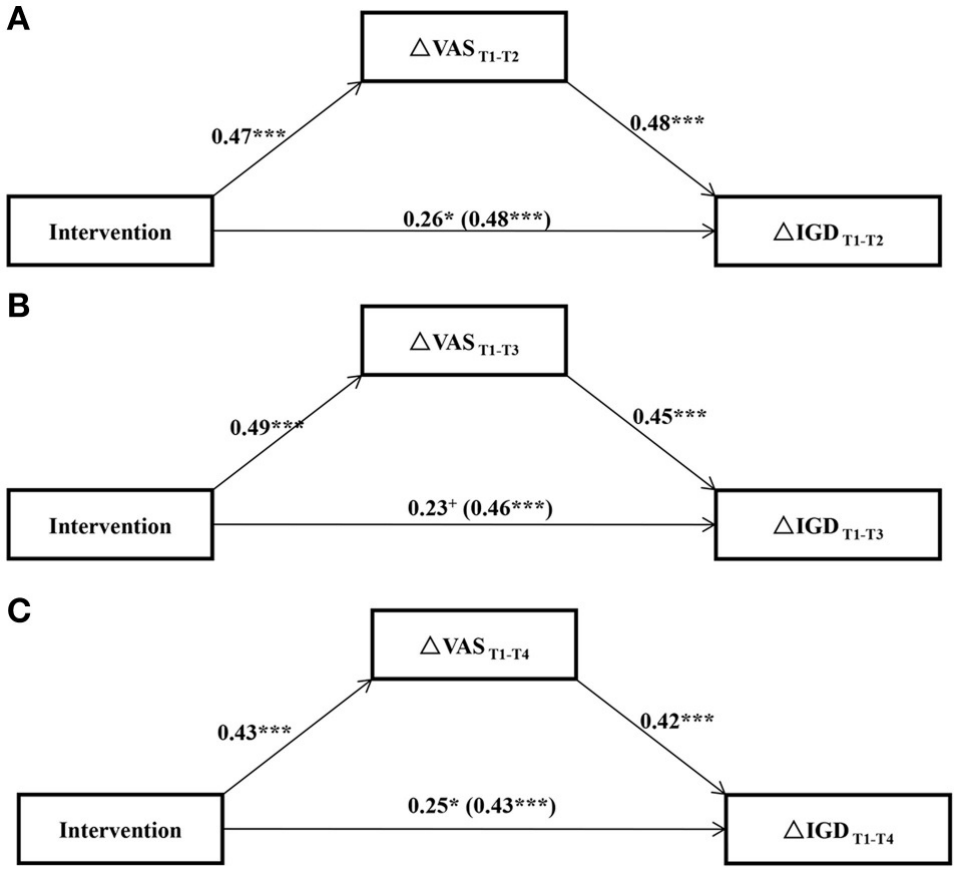
**The mediation effects of the craving-aimed intervention for the mitigation of IGD. (A)** The immediate effect (pre-post intervention) of the craving behavior intervention for the mitigation of IGD; **(B)** The short-term effect (pre-intervention and 3-month follow-up) of the intervention for the mitigation of IGD; **(C)** The long-term effect (pre-intervention and 6-month follow-up) of the intervention for the mitigation of IGD. ^+^*p* < 0.10; ^*^p < 0.05; ^***^p < 0.001.

Results from the tests of mediation effects showed that intervention and the change of self-reported craving could account for 41% (*F* = 20.83, *p* < 0.001), 36% (*F* = 16.51, *p* < 0.001), and 33% (*F* = 13.56, *p* < 0.001) of variance in the immediate, short-term, and long-term mitigation of IGD (see Figure 2). The mediation effects of craving-changing were 22.56, 22.05, and 18.06% separately, indicating partial mediating effects between the relation of intervention and IGD mitigation (see Figure 2).

### Explorative analyses for active ingredients of intervention

Since we hypothesized depression release, shifting young adults' fulfillment of psychological needs from the Internet to real life, coping skills learning of gaming-time management and impulse control as active ingredients of craving-aimed intervention, changes of these variables before and after intervention (at Time 1 and 2 for the CBI- group) were measured for both the intervention and control group. Significant group by assessment interactions were found for the depression and percentage of gaming hours separately [*F*_(1, 59)_ = 6.46, *p* < 0.05; *F*_(1, 59)_ = 5.79, *p* < 0.05]. Simple effect tests showed significant difference between the two time-point measurements in the intervention group [depression, *F*_(1, 57)_ = 34.95, *p* < 0.001; percentage of gaming hours, *F*_(1, 57)_ = 31.68, *p* < 0.001], whereas no significant differences were found in the control group. As for the fulfillment of psychological needs, significant decrease for the pre-post measurements displayed in the CBI+ group [*F*_(1, 57)_ = 7.81, *p* < 0.01] with no difference found in the CBI- group. Neither interaction nor simple effects were found in the scores of impulsivity (see Table 3).

**Table 3:** **Comparisons of active ingredients between the CBI+ and the CBI− group at T1 and T2 time points**.

**Variable**	**Group**	**T1**	**T2**	***F*****^a^**	***F*****^b^**
		***M (SD)***	***M (SD)***		
Depression	IGD+	33.23 (8.28)	26.57 (5.25)	6.46^*^	34.95^***^
	IGD−	30.83 (4.59)	29.31 (4.92)		0.82
	*t*	1.41	−1.88+		
Psy-needs	IGD+	135.05 (21.57)	124.50 (18.45)	1.68	7.81^**^
	IGD−	135.28 (18.98)	133.50 (25.12)		0.1
	*t*	−0.04	−1.53		
Gam%	IGD+	0.58	0.37	5.79^*^	31.68^***^
	IGD−	0.55	0.50		0.77
	*t*	0.53	−1.71+		
Impulsivity	IGD+	70.67 (6.90)	72.00 (6.32)	2.04	1.91
	IGD−	72.10 (5.93)	70.78 (7.25)		0.63
	*t*	−0.69	0.73		

* *p < 0.05*;

** *p < 0.01*;

*** p < 0.001;

F^a^ *, the effects of group × time points interaction*;

F^b^ *, the simple effects of time points in each group*.

To exam the hypothesis that effects of the carving intervention are mediated through major process variables, three hierarchical multiple regression analysis were performed related to immediate, short-term, and long-term effects separately (see Table 4). Before examining the mediating effects, intervention-related value changes (ΔX) were created for the depression release, the shifting of psychological needs fulfillment, percentage of gaming hours and impulsivity among the CBI+ group, by subtracting the post-intervention measures from the baseline (ΔX = X_T1_ − X_T2_). Craving-value changes for different time-points were created by subtracting the post-assessment time-point measures from the baseline (i.e., effects ΔX immediate = X_T1_ − X_T2_;ΔX short-term = X_T1_–X_T3_; ΔX long-term = X_T1_–X_T4_).

**Table 4:** **Regressions of change values of craving on changed values of intervention variables at post-intervention (ΔX = X_T1_−X_T2_)**.

	**Post-intervention**				**3-month-follow up**				**6-month-follow up**			
	**β**	***t***	***R*****^2^**	***F***	**β**	***t***	***R*****^2^**	***F***	**β**	***t***	***R*****^2^**	***F***
ΔDepression	0.36	2.67^*^	0.32	4.67^**^	0.27	1.70^+^	0.16	1.37	0.35	2.35^*^	0.31	3.94^**^
ΔPsy-needs	0.38	2.78^**^			0.16	1.01			0.31	2.08^*^		
ΔGam%	−0.02	−0.15			0.06	0.39			0.14	0.94		
ΔImpression	−0.05	0.35			0.02	0.10			−0.05	−1.19		

+ *p < 0.10*;

* *p < 0.05*;

** *p < 0.01*.

The pre-post changes of values for the depression release and shifting of psychological needs fulfillment could predict changes of craving (pre-post and pre-6 month follow-up measurements). The results indicated that 32% (pre-post) and 31% (pre-6 month follow-up) of the variance in the change in individuals' craving (*R*^2^ = 0.32, *R*^2^ = 0.31, respectively) were accounted for by improvements in the depression release and shifting of psychological needs fulfillment (see Table 4). Contrary to our hypothesis, percentage of gaming hours and impulsivity could not significantly predict the change of craving. Thus, only the depression and psychological needs are active ingredients of the craving behavior intervention.

Furthermore, three hierarchical multiple regression analyses were conducted to explore the partial effect of the active ingredients on mitigation of IGD when the effects of craving were controlled. The results revealed that the percentage of gaming hours and impulsivity could marginally predict the long-term change of IGD after controlling the effect the craving, while no significant effects were found for the depression release and fulfillment of psychological needs on mitigation of IGD. (see Table 5).

**Table 5:** **Regressions of changed values of IGD on changed values of measured variables at post-intervention (ΔX = X_T1_−X_T2_)**.

	**Post-intervention**						**3-month-follow up**						**6-month-follow up**					
	**β**	***t***	***R*****^2^**	***F***	***ΔR*****^2^**	***ΔF***	**β**	***t***	***R*****^2^**	***F***	***ΔR*****^2^**	***ΔF***	**β**	***t***	***R*****^2^**	***F***	***ΔR*****^2^**	***ΔF***
**STEP 1**																		
ΔVAS	0.60	4.83^***^	0.36	23.29^***^	0.36	23.29^***^	0.50	3.68^***^	0.25	13.56^***^	0.25	13.56^***^	0.46	3.17^**^	0.21	10.07^**^	0.21	10.07^**^
**STEP 2**																		
ΔVAS	0.44	3.09^**^	0.47	6.74^***^	0.11	2.03	0.46	3.14^**^	0.29	3.04^*^	0.04	0.55	0.45	2.69^*^	0.34	3.52^*^	0.13	1.70
ΔDepression	0.14	1.07					0.03	0.18					0.17	1.09				
ΔPsy-needs	0.18	1.37					0.08	0.54					−0.01	−0.09				
ΔGam%	0.16	1.32					0.02	0.15					−0.27	−1.83^+^				
ΔImpression	0.15	1.21					0.17	1.23					0.27	1.88^+^				

+ *p < 0.10*;

* *p < 0.05*;

** *p < 0.01*;

*** *p < 0.001*.

## Discussion

To our knowledge, the current study is the first to exam the effects as well as the active ingredients of craving-aimed intervention on problematic Internet gaming behaviors among individuals with IGD. As we have hypothesized, the CBI could effectively mitigate severity of IGD, and the value change of craving could partially explain the mitigation of IGD. This study further explored active ingredients of the craving-aimed intervention practice with the detection of depression and psychological needs accounted for the variance of the change in individuals' craving at post and 6 month follow-up measurements.

### Effects of craving regulation in ameliorating IGD

Craving may directly drive addictive behavior and relapse (Sinha, 2013). In the current study, significant decrease of craving and severity of IGD right after intervention were observed in the CBI+ group compared to CBI− group, which is consistent with previous studies on IGD, using pharmacological craving intervention (Han et al., 2010), and behavioral approach (Han et al., 2012; Zhang et al., 2016). This result is consistent with interventional studies in gambling disorder, that craving as a core component as well as a sensitive index could be reduced by the opioid antagonists (Grant et al., 2003; Brewer et al., 2008), while also by multiple behavioral therapies as a symptom of GD (Yip and Potenza, 2014). Further, in line with treatment studies on addictions (McCarthy et al., 2008; Piper et al., 2008; Subbaraman et al., 2013), and the assumption that craving may directly drive drug use or act as a precursor to relapse (Tiffany and Wray, 2012), a partial mediation effect of craving-reduction on the relationship of intervention and severity of IGD were demonstrated. That is, craving is not only the outcome of treatment, but also within a causal chain that plays a significant role in decreasing severity of IGD through CBI intervention. Our data therefore suggest validity of craving as an important treatment target in IGD treatment.

Further, 3- and 6-month follow-up were conducted tracking the short- and long-term effects of craving intervention on gaming addiction behavior, which previous studies were lack of, while interventional studies in GD also have this limitation. As hypothesized, a stable maintained effect of intervention was found in the CBI+ group, indicating that craving-aimed approach is very helpful in the intervention effect lasting. Similarly, evidence from substance use treatment practices also revealed the long-lasting effects of craving-regulation module on characteristics of addiction, ranging from 4 to 12 months (Piper et al., 2008; Witkiewitz and Bowen, 2010; Witkiewitz et al., 2011). Taken together, these results robustly support the assumption that craving reduction brought about through treatment might mediate long-term positive outcomes (Tiffany and Wray, 2012).

### Active ingredients of craving behavior intervention

Given that craving relief may be a valuable endpoint for addictions therapy (Addolorato et al., 2005; O'Brien, 2005), related induction elements may do as active ingredients affecting craving-reduction. The present study detected depression release and shifting of psychological needs satisfaction from Internet to real life significantly predict craving amelioration at both post-intervention and 6-month follow-up. Similarly, many previous researches have observed the mediation effect of craving amelioration on the relationship between negative emotion (e.g., depression) or stress and severity of addiction in substance use disorders (Witkiewitz and Bowen, 2010; Witkiewitz et al., 2011) and behavioral disorder (Chao et al., 2015). Recent review studies have hypothesized a framework of craving-as-emotion that various affective states can precipitate cravings and varies according to context (Heckman et al., 2013; Sayette, 2016). Emotion-regulation training in this study, mainly about depression, through relaxing and weakening its association with approaching behaviors to ameliorate craving, has been demonstrated both the immediate and long-lasting effects.

Improving young adults' satisfaction of psychological needs from real life instead of Internet is another active ingredient of this intervention practice. A previous study on adolescents with Internet addiction disorder has found the direct effect of the shifting fulfillment of psychological need on reduction of internet addiction (Liu et al., 2015). The current study further revealed the mechanism of psychological needs shift on IGD, that through increasing satisfaction of psychological needs from daily life activities (e.g., doing sports, adaptive interpersonal communicating) to reduce the dependence of Internet gaming and also craving for gaming, which further supported prior findings (Liu et al., 2012). Similarly, an interventional study on adolescents with IGD using family therapy approach aiming through enhancing family cohesion and affection has reduced craving and improved perceived family cohesion (Han et al., 2012). Taken together, online-gaming craving as a maladaptive desire may “hijack” basic processes of privileging certain stimuli and information that has adaptive values (Volkow and Baler, 2014; Sayette, 2016). Thus, behavior training on shifting needs fulfillment from Internet to real life may be a promising approach in craving amelioration, which warranted more research to support.

It is worth noting that the two ingredients were active in both immediate- after-intervention stage and 6-month-follow-up stage, except for the 3-month -follow-up stage. There are two potential interpretations for this. Firstly, addictive behavior cessation is a significant stressor causing mood ambivalent, with simultaneously strong behavior approaches and avoidance of doing so (Westbrook et al., 2013; Sayette, 2016). Then, based on the trans-theoretical model (TTM) of behavior change (Prochaska and Velicer, 1997), which assumes that health behavior change involves progress through five stages including pre-contemplation, contemplation, preparation action and maintenance stage, and the process of behavioral change is not linear but rather spiral, participants in the intervention group may present the transition from action to maintenance stage during the 3-month follow-up, and the progression appears to be unstable even regressing.

However, no predictive effects of percentage of gaming hours and impulsivity have been found either on craving or IGD amelioration. Gaming-time management as a coping strategy worked in improving participants' percentage time consuming on Internet gaming. Yet, no effects have been found of these two components on craving or IGD amelioration. Similarly, studies in GD performed into relationships among impulsivity, and treatment outcome to test the efficacy of serotonin reuptake inhibitors also found inconsistent efficacy (Leeman and Potenza, 2012). There are three alterations that may explain. Firstly, both bottom-up (emotion relief and fulfillment of psychological need) and top-down approaches (time management and impulse control) were used in the craving-aimed intervention practice. And the bottom-up path, according to the results, may be more dominant, which is consistent with some existing evidences (Tang et al., 2013; Westbrook et al., 2013). Secondly, pre-exit psycho-neural deficits of impulsivity in behavior addictions might weaken the efficacy of control-related training because the requirement of better self-control at baseline. Lastly, since impulsivity is a relative stable section of personality (Patton et al., 1995) and a subseptable trait of addiction, the effects through 6-week intervention may be too delicate to detect that more sensitive index to detect is needed (i.e., cognitive tasks or neural imaging strategies).

### Implications and limitation

Although many previous researches claimed that craving was the key element influencing addiction and relapse, this study was the first to clarify the effects and influencing mechanism of craving on IGD by interventional data, which provided persuasive evidence for developing theories about craving and IGD. Moreover, this study confirmed the core role of craving regulation in ameliorating IGD and indicated active ingredients of craving behavior intervention, which shed a light on the intervening direction of IGD in the future and would finally save more IGDs.

However, there are some limitations of this study. Since a group intervention approach was adopted, participants were assigned based on their schedule arrangement and willingness, that's, participants were not randomly assigned to the intervention or control group. Thus, the study lacks randomization. Although baseline comparisons between the two groups did not show a significant difference, indicating that the results of the study are reliable, this might still weaken the persuasion of the study. In addition, the subjects in this study were all undergraduate students, so the results might not be generalized to other populations. Therefore, we should pay attention to the generalization of intervention practices in future studies.

What's more, considering the fatigue, boredom, even wastage of the participants, we simplified the structure of the follow-up measurement, leading to the lack of active ingredients' tracking. It would be clearer if further clinical studies endeavored to keep tracking the change of active ingredients and its impact on craving and IGD. Additionally, the Cronbach's alpha of Impulsivity Scale Version 11 (BIS-11) in this study was low, which might be due to some culture shock that influenced the reliability of this instrumental conducted among Chinese undergraduate students; another interpretation might be the trait difference between IGD and other psychiatry disorders. Thus, a study to revise and determine the psychometric properties of the BIS-11 by using larger sample of Chinese undergraduate students (especially among college students with IGD) is necessary in the future.

## Author contributions

XF was responsible for the study concept and design. LL, CX, JL, and JZ contributed to the intervention practice and data acquisition. LD, LL, and XF assisted with data analysis and interpretation of findings. LL and LD drafted the manuscript. XF provided critical revision of the manuscript for intellectual content. All authors critically reviewed and approved the final version of the manuscript submitted for publication.

### Conflict of interest statement

The authors declare that the research was conducted in the absence of any commercial or financial relationships that could be construed as a potential conflict of interest.
