# Supplementary material for: Craving Behavior Intervention in Ameliorating College Students' Internet Game Disorder: A Longitudinal Study
Source: Front Psychol. 2017 Apr 10;8:526. doi: 10.3389/fpsyg.2017.00526 (PMC5385373; doi:10.3389/fpsyg.2017.00526)
Supplement: Supplementary file 1 [file Presentation1.PDF]

Craving behavior intervention in ameliorating college students' Internet game disorder: A longitudinal study

Supplementary information

Supplemental methods

Participants continued

Tobacco-use characteristics were assessed using the Fagerstrom Test for Nicotine Dependence (FTND) (Fagerstrom, 1978), and nicotine-dependent individuals were excluded (i.e., individuals with an FTND score ≥6) (Fagerstrom et al., 1990). Alcohol consumption was assessed using the Alcohol Use Disorder Identification Test (AUDIT-C) (Bush et al., 1998), and participants with AUDIT-C scores ≥ 5 (Dawson et al., 2005) were instructed to complete the Michigan Alcoholism Screening Test (MAST) (Selzer, 1971) for further screening. Individuals with a score ≥ 6 on the MAST were excluded for alcohol dependence.

Intervention

Forty-four of the IGD participants under intervention were divided into 5 groups with 8-10 persons each group. Among them, 3 groups were intervened by a couple of therapists, while the other 2 group were intervened by another couple of therapists.

Supplemental results

Comparison of intervention effects of two groups of therapists

Results from repeated measures ANOVA (see Table S1) showed no therapists by assessment (pre-post-intervention/assessment) interaction for VAS, severity of IGD (score of POGUS), also no significant main effects of both therapists and assessment were found (see Table S1). These results demonstrated the homogeneity of the two groups of therapists.

Table S1

Comparisons of measured variables between the two groups of therapists at T1, T2, T3 and T4.

**Variable**	**Group**	**T1**	**T2**	**T3**	**T4**	***F*** [a]
		M (SD)	***M (SD)***	***M (SD)***	***M (SD)***	

IGD	IGD+	76.46<br>（12.78）	56.54<br>（6.98）	51.50<br>（11.85）	51.82<br>（12.69）	0.60
	IGD-	79.07<br>（9.68）	55.36<br>（11.44）	55.43<br>（12.59）	55.93<br>（10.64）	
	***F*[b]**	0.44	0.17	0.96	1.06	
VAS	IGD+	3.52<br>(1.23)	2.41<br>(1.06)	2.30<br>(1.01)	2.33<br>(0.94)	0.52
	IGD-	2.79<br>(1.55)	3.32<br>(1.38)	3.74<br>(1.69)	3.44<br>(1.54)	
	***F*[b]**	3.27	1.34	0.22	3.94	

F ^{a}: the effects of therapist-group × time points interaction;

F ^{b}: the simple effects of therapist-group in each time points.

References

Bush K, Kivlahan DR, McDonell MB, Fihn SD, Bradley KA (1998) The AUDIT alcohol consumption questions (AUDIT-C): an effective brief screening test for problem drinking. Archives of internal medicine 158:1789-1795.

Dawson DA, Grant BF, Stinson FS, Zhou Y (2005) Effectiveness of the derived Alcohol Use Disorders Identification Test (AUDIT-C) in screening for alcohol use disorders and risk drinking in the US general population. Alcoholism: Clinical and Experimental Research 29:844-854.

Fagerstrom KO (1978) Measuring degree of physical dependence to tobacco smoking with reference to individualization of treatment. Addictive behaviors 3:235-241.

Fagerstrom KO, Heatherton TF, Kozlowski L (1990) Nicotine addiction and its assessment. Ear Nose Throat J 69:763-765.

Selzer ML (1971) The Michigan alcoholism screening test: the quest for a new diagnostic instrument. American Journal of Psychiatry 127:1653-1658.
